# Pain severity predicts depressive symptoms over and above individual illnesses and multimorbidity in older adults

**DOI:** 10.1186/s12888-017-1334-y

**Published:** 2017-05-04

**Authors:** Louise Sharpe, Sarah McDonald, Helen Correia, Patrick J. Raue, Tanya Meade, Michael Nicholas, Patricia Arean

**Affiliations:** 1 School of Psychology, University of Sydney, Sydney, Australia; 2 Department of Psychology, Macquarie University, Sydney, Australia; 3 School of Psychology, Murdoch University, Perth, Australia; 4 Psychiatry and Behavioral Sciences Division of Population Health, AIMS Centre University of Washington, Seattle, USA; 5 School of Social Sciences and Psychology, University of Western Sydney, Sydney, Australia; 6 School of Medicine, University of Sydney, Sydney, Australia; 7 Pain Management Research Institute, University of Sydney at Royal North Shore Hospital, Sydney, Australia

**Keywords:** Depression, Chronic illness, Pain, Multi-morbidity, Older adults

## Abstract

**Background:**

Multi-morbidity in older adults is commonly associated with depressed mood. Similarly, subjective reports of pain are also associated with both physical illness and increased depressive symptoms. However, whether pain independently contributes to the experience of depression in older people with multi-morbidity has not been studied.

**Methods:**

In this study, participants were 1281 consecutive older adults presenting to one of 19 primary care services in Australia (recruitment rate = 75%). Participants were asked to indicate the presence of a number of common chronic illnesses, to rate their current pain severity and to complete the Geriatric Depression Scale.

**Results:**

Results confirmed that the number of medical illnesses reported was strongly associated with depressive symptoms. Twenty-six percent of participants with multi-morbidity scored in the clinical range for depressive symptoms in comparison to 15% of participants with no illnesses or a single illness. In regression analyses, the presence of chronic pain (*t* = 5.969, *p* < 0.0005), diabetes (*t* = 4.309, *p* < 0.0005), respiratory (*t* = 3.720, *p* < 0.0005) or neurological illness (*t* = 2.701, *p* = 0.007) were all independent contributors to depressive symptoms. Even when controlling for each individual illness, and the overall number of illnesses (*t* = 2.207, *p* = 0.028), pain severity remained an independent predictor of depressed mood (F change = 28.866, *p* < 0.0005, *t* = 5.373, *p* < 0.0005).

**Conclusions:**

Physicians should consider screening for mood problems amongst those with multi-morbidity, particularly those who experience pain.

## Background

Multi-morbidity is defined as the presence of two or more chronic illnesses. Multi-morbidity is becoming an increasing problem with nearly a quarter of all primary care patients experiencing two or more medical conditions [1]. Moreover, amongst older people multi-morbidity is the norm (e.g., [1–4]). Estimates of multi-morbidity indicate that over 75% of adults older than 75 years presenting to primary care services have two or more chronic illnesses [3]. There is a considerable literature that confirms that the presence of multi-morbidity is associated with increased depressive symptoms in older adults (e.g., [1, 2, 5–9]). Although relatively few studies have investigated the relationship between multi-morbidity and diagnosed depressive disorders, the research is consistent in indicating that multi-morbidity is associated with a greater risk of a depressive disorder (e.g., [10, 11]).

Older adults with multi-morbidity also live with persistent pain [3, 12]. For example, estimates indicate that at least 60% of patients over 65 years with either heart failure or diabetes report clinically significant pain [13]. Just as multi-morbidity confers risk of developing clinically significant symptoms of depression in older adults, so too does the experience of pain [14]. While most of the research on the relationship between pain and mood involves self-report of depressive symptoms, a strong relationship between major depressive episode and chronic pain has also been confirmed [15].

While it is known that pain in the context of multi-morbidity is associated with poorer health-related quality of life and use of more health care resources in older adults [16–18], whether pain makes an independent contribution to low mood in this group remains unknown. In addition, most of the literature on the relationship between pain and depression has been conducted in samples of patients in which pain is the cardinal symptom of their health problems [e.g. chronic musculoskeletal pain [14], rheumatoid arthritis [19], fibromyalgia [20]. However, recent research indicates that the presence of pain is associated with depressed mood in a range of other illnesses, where pain is one symptom but not necessarily the cardinal symptom for which the patient is seeking treatment. For example, in patients following spinal cord injury, the experience of chronic widespread pain is associated with psychiatric symptoms [21]. Similar findings have been reported in patients with multiple sclerosis [22], cancer [23] and arthritis [24].

The primary aim of the current study is to explore the independent relationship between pain and depression in the context of multi-morbidity. In line with previous literature (e.g., [1, 2, 5–9]) we hypothesize that the number of illnesses experienced by older adults will be associated with increased depressive symptoms. Also in accordance with prior findings [13, 14] we hypothesize that the proportion of patients who report clinically significant depressive symptoms and at least moderate pain will be higher amongst those with multi-morbidity than those without. Finally, we predict that, after controlling for demographic variables, the presence or absence of individual illnesses and the number of illnesses, subjective pain will contribute independently to depressed mood.

## Method

### Participants

Participants were drawn from a large clinical trial comparing two psychological treatments for depression for patients with multi-morbidity [25]. Administrative staff in primary care practices or the researchers approached consecutive attendees 65 years or over in GP waiting rooms between 20th March 2013 and 6th May 2014. Seventeen hundred and seventeen completed a self-report screening questionnaire (“the screener”), of which 436 were blank, leaving 1281 screeners. We did not replace missing data since we had sufficient power for our analyses due to the large dataset and therefore the regression analyses are reported based on 887 participants who completed every question. The research was approved by the Human Research Ethics Committee (HREC) of The University of Sydney.

### Measures

In order to maximize the recruitment rate, the self-report screening questionnaire was kept as brief as possible and fit on a double-sided single sheet of paper.

#### Demographic details

Participants were asked to indicate their gender, age and marital status.

#### Illnesses

The screener included the following illnesses and asked participants to indicate whether or not they had a particular illness: respiratory disease, heart condition, diabetes, vascular disease, neurological condition, arthritis, kidney or urinary tract disease, liver disease, cancer, infectious disease, osteoporosis, sensory problem or chronic pain. These categories were chosen based on the disease categories in the cumulative illness rating system [26]. Participants were also given the option of nominating an illness that was not specified as “other”, which were then categorized according to the preceding illnesses (where possible) by the researchers. We specifically did not include conditions that confer risk for the development of chronic health problems, but are largely asymptomatic in and of themselves (such as hypertension, hypercholesterolism or obesity).

#### Depression

The Brief Geriatric Depression Scale (GDS; [27]) is a reliable, well validated 15 item questionnaire specifically developed to assess depression amongst older adults. The questions require a yes or no response and ask participants about a range of depressive symptoms. Research indicates that the 15 item GDS at a cut-off score of 5 on GDS has good specificity and sensitivity in detecting cases of clinical depression, as measured against the gold standard of a diagnostic interview [28]. Importantly, the GDS has been found to be robust in people with mild cognitive impairment [29]. The Cronbach alpha in this sample was 0.80.

#### Pain

A visual analogue scale (VAS) was provided for participants to indicate where on a 10 cm line their current pain was. The VAS was anchored with “No pain at all” at one end and “Worst pain imaginable” at the other end. We chose to assess current pain, as research indicates considerable recall bias and subsequent unreliability when asking participants to average pain across previous time periods [30]. While most of the validation studies for the VAS are with younger adults, self-report using VAS or similar rating scales is nonetheless a valid method of assessing current pain in older adults [31]. For the purposes of this study, we classified a score of 4 or greater as indicating moderate to severe pain. This is consistent with previous research that found moderate pain was rated by patients as having a mean of 49 on a 100 cm VAS, with a standard deviation of 17 [32]. Hence, pain ≥4/10 indicates those within half a standard deviation of what patients considered to be at least moderate pain.

### Procedure

Participants were asked to complete a brief (one page, double-sided) self-report screening questionnaire (“the screener”) while waiting for the GP. The screener asked basic demographic details, the presence or absence of various illnesses, subjective pain severity on a visual analogue scale and depressive symptoms (see Measures). Participants were asked to place the completed screener in a locked box located in the practice, which was collected weekly by the research team. Those who preferred not to complete the screener were also asked to leave the blank form in the locked box to allow for accurate estimates of the recruitment rate.

### Analyses

In order to investigate the nature of the relationships between number of illnesses, pain severity and depressive symptoms, correlational analyses were initially planned in order to determine the bivariate correlations between pain, various illnesses and depression. Further, to ascertain whether there were significant differences in depressive symptoms and pain severity dependent upon number of illnesses, two one-way ANOVAs were planned with number of illnesses as the independent variable.

A series of independent sample *t*-tests were then conducted to determine whether individuals with particular types of illnesses reported higher levels of depressive symptoms or pain than individuals without any illnesses. There were some minor violations of the data for depression and pain from the normality assumptions that underlie parametric statistics. For this reason, we replicated the analyses using non-parametric statistics and the pattern of results was unchanged. We have only reported parametric analyses below.

Finally, a hierarchical multiple regression equation was conducted to determine whether subjective reports of pain contributed over and above individual illnesses to depressive symptoms. Demographic variables associated with depressive symptoms in univariate analyses were added to the first step of the model. We intended to take a conservative approach by controlling for each individual illness separately and then adding the number of illnesses. So, on the second step we added the individual illnesses for which significant differences emerged between those who did and did not have the illness on either depressive symptoms or pain severity. On the third step of the equation, we added the number of illnesses (0–5+). On the final step of the equation, pain severity was added.

## Results

### Demographic characteristics

The mean age of the sample was 75 years (SD = 7.9) and 55% of the sample were female. Fifty-eight percent of the sample were married or living in a de facto relationship. Arthritis was the most commonly reported illness (50%), followed by cardiovascular problems (29%), osteoporosis (20%), diabetes (19%) and chronic pain (18%). Of the 1239 participant who completed all the illness questions, 181 (14.6%) did not report a physical illness and 360 people (28.1%) had only one illness. Nearly one quarter (*n* = 360, 24%) had two illnesses, and the remainder had three (*n* = 204, 15.9%), four (*n* = 106, 8.3%) or five or more illnesses (*n* = 81, 7.5%). Both the median and mean number of illnesses was 2.0 (SD = 1.5). Across the entire sample, the average level of depressive symptoms, according to the GDS was 2.54 (SD = 2.71) and 22% of the sample fell above the clinical cut-off point for likely depression (GDS ≥ 5). The average pain severity was mild 2.03 (SD = 2.55), however, 36% of the sample rated their pain as at least moderate in severity (i.e. pain rating ≥ 4). See Table 1 for a summary of demographic details.

**Table 1:** Demographic details of the sample, including number (and Percentage) of patients reporting each illness

	Mean (Standard Deviation)	Number (Proportion)
Age	75.46 (7.4)	
Gender		563/1264 Male (44.5%)
Marital status		718/1239 married^a^ (57.9%)
Respiratory disease		217/1241 (17.5%)
Heart condition		358/1241 (28.8%)
Diabetes		238/1241 (19.2%)
Vascular disease		125/1240 (10.1%)
Neurological condition		71/1241 (5.7%)
Arthritis		622/1241 (50.1%)
Kidney or urinary tract disease		106/1241 (8.5%)
Liver disease		26/1241 (2.1%)
Cancer		169/1241 (13.6%)
Infectious disease		23/1241 (1.9%)
Osteoporosis		253/1241 (20.4%)
Chronic pain		227/1241 (18.3%)
Multi-morbidity		698/1239 (56.3%)
Depression (GDS)	2.54 (2.7)	255/1136 > 5 on GDS (22.4%)
Pain level	2.04 (2.6)	466/1216 ≥ 4/10 on VAS (38%)

Key: *GDS* Geriatric Depression Scale, *VAS* visual analogue scale; ^a^married includes married or de facto

Because there was a relatively large amount of individual missing data points, we compared those participants who completed all aspects of the assessment with the rest of the sample. Participants who completed all sections of the form versus those that did not did not differ in terms of their age (*t* = 0.002, *p* = 0.998, 95% CI = [−1.048, 1.050]), the mean number of illnesses (*t* = 0.058, *p* = 0.954, 95% CI = [−0.223, 0.236]) or their level of pain (*t* = −0.664, *p* = 0.507, 95% CI = [−1.946, 0.962]). However, there was a significant difference between the groups for depressive symptoms (*t* = −2.021, *p* = 0.045, 95% CI = [−1.067, −0.013]), which indicated that those who did not complete all sections were more likely to have higher levels of depressive symptomatology.

### Illness and depressive symptoms

**Fig. 1:**
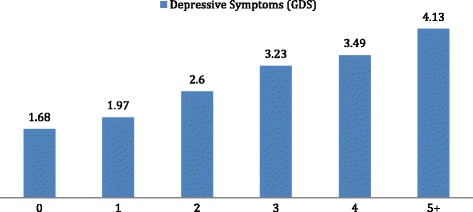
The number of depressive symptoms by the number of illnesses

There was a positive correlation between the number of illnesses reported and severity of depressive symptoms (*r* = 0.26, *p* < 0.0005). Indeed, there was a significant difference in depressive symptoms depending upon the number of medical illnesses (1 vs 2 vs 3 vs 4 vs 5+) reported (F_(5,1049)_ = 15.674, *p* < 0.0005). For those with multi-morbidity, 26% fell in the likely depressed range on the GDS compared to 15% of those with a single or no physical illness χ^2^(1, 1112) = 17.343, *p* < .0001 (See Fig. 1). Importantly, of those who had multi-morbidity *and* moderate to severe pain, 34% scored in the clinical range for depression.

Independent samples *t*-tests were conducted for those illnesses where at least 50 participants endorsed the presence of that category of illness. The illnesses associated with higher levels of depressive symptoms were: arthritis (*t* = 2.467, *p* < 0.05, 95% CI = [0.018, 0.161]), chronic pain (*t* = 4.114, *p* < 0.0001, 95% CI = [0.067, 0.191]), diabetes (*t* = 2.148, *p* < 0.05, 95% CI = [0.006, 0.125]), kidney disease (*t* = 2.267, *p* < 0.05, 95% CI = [0.007, 0.099]), neurological disorders (*t* = 2.754, *p* < 0.01, 95% CI = [0.016, 0.095]), and respiratory disease (*t* = 3.220, *p* = 0.001, 95% CI = [0.039, 0.160]). Table 2 gives descriptive statistics for patients with and without particular illnesses for depressive symptoms and pain level.

**Table 2:** Level of depressive symptoms (according to the Geriatric Depression Scale) for patients reporting each illness

	Illness not present	Illness present
	Mean (Standard Deviation)	Mean (Standard Deviation)
***Respiratory disease	2.36 (2.6)	3.41 (2.9)
Heart condition	2.5 (2.8)	2.69 (2.6)
***Diabetes	2.38 (2.7)	3.3 (2.8)
Vascular disease	2.51 (2.7)	2.99 (2.8)
**Neurological condition	2.48 (2.7)	4.00 (3.4)
***Arthritis	2.25 (2.6)	2.87 (2.8)
*Kidney or urinary tract disease	2.49 (2.7)	3.23 (2.5)
Cancer	2.52 (2.7)	2.77 (2.5)
Osteoporosis	2.41 (2.6)	3.09 (3.0)
***Chronic pain	2.26 (2.5)	3.96 (3.4)
***Multi-morbidity	1.87 (2.4)	3.08 (2.8)
***Pain level	2.07 (2.4)	3.39 (3.4)

Key. *p < 0.05; **p < 0.01; ***p < 0.001

### Illness and pain

There was a positive correlation between pain severity and number of illnesses (*r* = 0.357, *p* < 0.0005) and between pain severity and depression (*r* = 0.272, *p* < 0.0005). There was also a significant difference in pain severity dependent upon the number of illnesses (F_(5,1036)_ = 35.011, *p* < 0.0005). For those with multi-morbidity, 46% reported moderate to severe pain (i.e. pain severity ≥4), whereas this was true of only 25% of those with no illness or a single illness.

In terms of specific illnesses, those with the following illnesses reported significantly more pain than those without an illness: arthritis (*t* = 13.758, *p* < 0.0001, 95% CI = [0.338, 0.450]), chronic pain (*t* = 12.549, *p* < 0.0001, 95% CI = [0.217, 0.298]), kidney disease (*t* = 2.769, *p* = 0.006, 95% CI = [0.013, 0.079]), osteoporosis (*t* = 6.098, *p* < 0.0001, 95% CI = [0.098, 0.191]) and respiratory disease (*t* = 2.701, *p* = 0.007, 95% CI = [0.017, 0.108]). Means and standard deviations presented in Table 3.

**Table 3:** Pain severity (according to the Visual Analogue Scale) for Patients Reporting Each Illness

	Illness Not Present	Illness Present
	Mean (Standard Deviation)	Mean (Standard Deviation)
*Respiratory disease	1.95 (2.5)	2.43 (2.5)
Heart condition	2.08 (2.6)	1.91 (2.4)
Diabetes	2.00 (2.5)	2.19 (2.7)
Vascular disease	1.98 (2.5)	2.49 (2.8)
Neurological condition	2.01 (2.5)	2.45 (2.7)
***Arthritis	1.05 (1.9)	3.02 (2.7)
**Kidney or urinary tract disease	1.97 (2.5)	2.71 (2.6)
Cancer	1.99 (2.6)	2.29 (2.3)
***Osteoporosis	1.81 (2.5)	2.93 (2.7)
***Chronic pain	1.42 (2.1)	4.73 (2.7)
***Multi-morbidity	1.07 (2.0)	2.78 (2.7)
***Depressive symptoms	1.76 (2.3)	2.82 (3.0)

Key. *p < 0.05; **p < 0.01; ***p < 0.001

### Regression analyses

A series of multiple regression analyses were run to determine whether subjective reports of pain contributed over and above individual illnesses to depressive symptoms. As both pain and depression were not normally distributed, these variables were transformed using a square root transformation and subsequently met the statistical assumptions for the regression. The demographic variables that were correlated with depression were added to the first step of the model. These accounted for 2% of the variance in depressive symptoms (F _3887_ = 5.280, *p* = 0.001). The following illnesses (present or absent) were entered into the second step of the regression equation: arthritis, chronic pain, diabetes, kidney or urinary tract disease, neurological conditions, osteoporosis and respiratory disease. These illnesses accounted for an additional 11% of the variance in depressive symptoms (F_7,877_ change = 7.219, *p* < 0.0005). On the third step, the number of illnesses added a further 1% to the predictive value of the regression equation. (F change_1,876_ = 4.872, *p* = 0.028). On the final step, however, pain severity still accounted for an additional 2.4% of the variance in depressive symptoms (F change_1,875_ = 28.866, *p* < 0.0005).

As can be seen in Table 4, in terms of individual predictors, marital status was the only demographic variable associated with independent variance in depressive symptoms. On step 2, chronic pain, diabetes, neurological and respiratory disease all contributed independently to depressive symptoms. On step 3, number of illnesses also contributed a small, but significant amount to the variance in depressive symptoms. However, even once controlling for all these factors, pain severity remained a significant predictor (Beta = 0.191; *t* = 5.373, *p* < 0.0005).

**Table 4:** Hierarchical regression equation to predict depressive symptoms according to the Geriatric Depression Scale

Step of the model	R^2^ change	F change	Significance
Demographic variables	0.018	5.280	*p* = 0.001
Individual Illnesses	0.109	7.219	*p* < 0.0005
Number of illnesses	0.05	4.872	*p* = 0.028
Pain severity	0.028	28.866	*p* < 0.0005
Step 2: Individual Predictors	Beta	*t*-value	Significance
Marital status	.063	2.739	*p* = 0.006
Chronic pain	.468	5.969	*p* < 0.0005
Diabetes	.326	4.309	*p* < 0.0005
Neurological disease	.364	2.708	*p* = 0.007
Respiratory condition	.289	3.720	*p* < 0.0005
Step 3: Individual predictors	Beta	t-value	Significance
Number of illnesses	.137	2.207	*p* = 0.028
Step 4: Individual predictors	Beta	t-value	Significance
Pain severity	.191	5.373	*p* < 0.0005

## Discussion

The primary aim of the present study was to determine whether subjective reports of pain were associated with depressive symptoms in a group of older adults with multi-morbidity. Our results supported the hypotheses. That is, both depressive symptoms and subjective pain were associated with the number of illnesses that participants reported. Further, rates of clinically significant depressive symptoms were more common amongst those with multi-morbidity (26%) compared to those without illness or with a single illness (15%). The pattern of results was similar for clinically significant levels of pain. That is, 46% of those with multi-morbidity reported moderate to severe levels of pain (VAS ≥ 4), in comparison with 25% of those without multi-morbidity. As we predicted, there were significant associations between pain and depressive symptoms. Importantly, in older adults depressive symptoms remained associated with pain, even after controlling for the presence of absence of individual illnesses and the number of illnesses.

The findings from the present study are consistent with the available literature in neurological disorders [33], respiratory disease [34], diabetes [35] and chronic pain [14]. Given the strong relationship between pain and depressed mood in other studies (e.g., [19, 22, 23, 36]), it is perhaps unsurprising that pain predicted depressive symptoms in the context of multi-morbidity as well. This finding does have important clinical implications. Firstly, there is considerable evidence that both depression [37] and pain [38] are undertreated amongst older people. These data suggest that for older patients with multi-morbidity screening for pain and risk of depression would be worthwhile. It is well documented that there are numerous barriers to accessing appropriate care for older people with depression [39] and this is also true of pain [38].

The identification of risk of depressive disorders in patients with multi-morbidity is important because research clearly shows that depression is associated with poorer health-related quality of life [11], more hospitalizations [40], poorer health outcomes [41] and meta-analyses indicate greater mortality from conditions such as stroke [42] and diabetes [42]. Although the association with pain and multi-morbidity is less well studied, pain and depression have been shown to have a reciprocal relationship [43]. Therefore, there is a good rationale to identify and treat these problems in older people with multi-morbidity. However, medical management of these symptoms can be more complex in patients with multi-morbidity [38].

Patients with multi-morbidity are typically on a number of medications to try and manage their underlying chronic illnesses. Hence, the most common treatments prescribed in general practice for depression and pain (i.e. anti-depressants and analgesia) are often contra-indicated in these patients [44–46]. For example, there is evidence from population cohort studies that the prescription of anti-depressant medication to elderly people with depression is associated with an increased risk of adverse events, including falls and completed suicides [47, 48]. Recent data also questions the safety of opiates in the elderly [46]. Indeed, the perceived lack of appropriate medical options for these symptoms is a potential barrier to the identification and management of depression and pain in this population. However, there are studies in the primary care context that have found that collaborative care provided to patients with physical illnesses (cardiovascular and diabetes) that implement interventions consistent with patient guidelines results in superior patient outcomes [49]. Therefore, it is imperative to establish evidence-based practice for this group of patients with complex needs, which minimize adverse events but maximize efficacy.

As with younger adults, there is now good meta-analytic evidence that psychosocial interventions are effective in the management of late-life depression [50, 51], with available evidence suggesting that psychosocial treatments appear to be as effective as anti-depressant medications [52]. However, there is some evidence of smaller effect sizes in patients with comorbid physical illness [53]. Nonetheless, there is good reason to think that if depression is identified in patients with multi-morbidity, then psychosocial interventions can be effective, although they may need to be adapted to the physical abilities of the patients [53].

Similarly, there has been recent interest in extending successful approaches to pain management to older people [54, 55]. Trials have shown that pain management programs based on cognitive behavioural approaches are effective in improving disability and mood in patients with clinically significant pain problems. A recent review of the literature indicates that there is sufficient evidence to judge cognitive-behavioural based pain management programs to be effective with older adults [56]. Participants in these studies typically have a high mean number of illnesses (e.g. TG Mayer, BL Towns, R Neblett, BR Theodore and RJ Gatchel [21] had a mean of three illnesses) and therefore, there is good reason to believe that these approaches would result in significant patient gain for those with complex needs, such as multi-morbidity. This is especially true of the older (> 75 years) and more frail elderly, where pain has been shown to be a particularly important challenge [57].

This is an area that will become of increasing importance in the coming decades. Not only is the population ageing and consequently older adults are living longer with more chronic conditions. However, there is also evidence that multi-morbidity is becoming more common in younger age groups, as the population engages in less healthy lifestyles and obesity rates continue to rise. There has recently been a call to shift from a model of treating individual illnesses to studying the efficacy of interventions in the context of complex health needs, which are becoming increasingly common [1, 58]. The results of this study support the need to test the efficacy of known efficacious treatments, specifically in patients with multi-morbidity.

Clearly this study provides only a snapshot of the cross-sectional relationships between variables and there are limitations to the methodology that we used. Firstly, since it is cross-sectional, we do not know whether pain influences depressive symptoms or vice versa. In reality, it is likely that this relationship is reciprocal [59]. Secondly, use of a cross-sectional method restricted collection of information about pain levels over a longer time period. Thirdly, in order to maximize our recruitment, we could only measure a small number of variables and so it is possible that there are important covariates that we were unable to assess. These variables could possibly have had relationships with depression. Additionally, illnesses were self-reported on the screener and thus without medical confirmation diagnoses the reliability of this information may be compromised and we can only make claims about the presence or absence of illnesses not illness severity. It is possible that those who were most depressed were more likely to endorse more illnesses and this may have affected results. Finally, we also did not have exclusion criteria and invited all people over 65 to take part in the study. While this increases the generalizability of our results to primary care settings, it meant that we were unable to screen out people with cognitive impairment, which may have reduced the reliability of the data particularly as it was based on self-report. In fact, those who did not complete all segments of the screener were more likely to be depressed, which is also reported for patients who have mild cognitive impairment [60]. Although in all other ways, the participants that did and did not complete the entire screener appeared similar, it is possible that this reduces the validity of our results. However, in large samples of this nature, the reliability of responses is largely unavoidable.

## Conclusions

These limitations notwithstanding, this study contributes to a body of literature that confirms that multi-morbidity is indeed associated with depressive symptoms and pain [2, 3]. Our results confirm that the more illnesses a person experiences, the more depressive symptoms they report and the higher the levels of their subjective pain. Importantly, one-third of participants who had both two or more physical illnesses and a moderate to severe level of pain, scored in the clinical range of depressive symptoms on the GDS. These results highlight the importance of screening patients with multi-morbidity for both pain and risk of depression and ensuring that they have access to effective interventions. There is a growing evidence base for the treatment of late-life depression and pain in older adults, but more research is needed to investigate these approaches in patients with complex health needs.

## Abbreviations

GDS: Geriatric depression scale
VAS: Visual analogue scale
